# Performance Comparison of Fuzzy ARTMAP and LDA in Qualitative Classification of Iranian *Rosa damascena* Essential Oils by an Electronic Nose

**DOI:** 10.3390/s16050636

**Published:** 2016-05-04

**Authors:** Abbas Gorji-Chakespari, Ali Mohammad Nikbakht, Fatemeh Sefidkon, Mahdi Ghasemi-Varnamkhasti, Jesús Brezmes, Eduard Llobet

**Affiliations:** 1 Department of Mechanical Engineering of Biosystems, Urmia University, Urmia 5756151818, Iran; a.nikbakht@urmia.ac.ir; 2 Research Institute of Forests and Rangelands, Tehran 1496813111, Iran; sefidkon@rifr-ac.ir; 3 Department of Mechanical Engineering of Biosystems, Shahrekord University, Shahrekord 8818634141, Iran; ghasemymahdi@gmail.com; 4 Department of Electronic, Electrical and Automatic Control Engineering, Universitat Rovira i Virgili, Tarragona 43007, Spain; jesus.brezmes@urv.cat

**Keywords:** Fuzzy ARTMAP, LDA, electronic nose, *Rosa damascene*, essential oil, classification

## Abstract

Quality control of essential oils is an important topic in industrial processing of medicinal and aromatic plants. In this paper, the performance of Fuzzy Adaptive Resonant Theory Map (ARTMAP) and linear discriminant analysis (LDA) algorithms are compared in the specific task of quality classification of *Rosa damascene* essential oil samples (one of the most famous and valuable essential oils in the world) using an electronic nose (EN) system based on seven metal oxide semiconductor (MOS) sensors. First, with the aid of a GC-MS analysis, samples of *Rosa damascene* essential oils were classified into three different categories (low, middle, and high quality, classes C1, C2, and C3, respectively) based on the total percent of the most crucial qualitative compounds. An ad-hoc electronic nose (EN) system was implemented to sense the samples and acquire signals. Forty-nine features were extracted from the EN sensor matrix (seven parameters to describe each sensor curve response). The extracted features were ordered in relevance by the intra/inter variance criterion (Vr), also known as the Fisher discriminant. A leave-one-out cross validation technique was implemented for estimating the classification accuracy reached by both algorithms. Success rates were calculated using 10, 20, 30, and the entire selected features from the response of the sensor array. The results revealed a maximum classification accuracy of 99% when applying the Fuzzy ARTMAP algorithm and 82% for LDA, using the first 10 features in both cases. Further classification results explained that sub-optimal performance is likely to occur when all the response features are applied. It was found that an electronic nose system employing a Fuzzy ARTMAP classifier could become an accurate, easy, and inexpensive alternative tool for qualitative control in the production of *Rosa damascene* essential oil.

## 1. Introduction

Essential oils are highly concentrated, volatile, hydrophobic mixtures of chemicals extracted from plants [1]. These materials usually consist of a complex mixture from tens to hundreds of low molecular weight terpenoids. Due to their flavor and fragrance properties, essential oils have many applications in several fields including the food industry (e.g., soft drink, food additive, and confectionary), the cosmetic industry (e.g., perfume, skin, and hair care products) and the pharmaceutical industry for their anti-HIV (Human Immunodeficiency Virus), anti-bacterial, anti-oxidation, and sedation properties [2,3,4]. Despite their wide range of applications, about 90% of global essential oil production is consumed by the flavor and fragrance industry, including perfumes and foods. Indeed, the higher growths in the market of essential oils and extracts, estimated at more than 5% per year, are foreseen in flavor and fragrance applications [1]. Also, the global trade of essential oils was valued to be around five billion dollars in 2011 while a 11.67 billion dollar value is expected for this market by 2022 [5].

The most aromatic rose species, scientifically named as *Rosa damascena* Mill., known as “Gol-e-Mohammadi” in Persian, is cultivated extensively in Iran, Turkey, and Bulgaria [6]. The essential oil of *Rosa damascene* is the most market valuable essential oil in the world ($7,500/kg), and this is why it is nicknamed “liquid gold” [7]. Its high price is due to the large amounts of rose petals typically needed to extract adequate enough amounts of oils. For example, the production of 1 kg of rose oil requires 4000 kg of rose petals [1]. This essential oil is vastly employed in the above mentioned industries. The quality control of essential oils has a very important role in the industrial processes related to the development of flavors and fragrances.

One quality monitoring method is the use of instrumental techniques such as gas chromatography (GC), GC coupled with mass spectrometry (GC/MS), high performance liquid chromatography (HPLC), and thin layer chromatography (TLC), which are objective and precise but expensive, destructive, time-consuming, and need to be performed by well-trained operators [8,9]. Therefore, the development of easy and low cost methods similar to those obtained by electronic noses (ENs) could be of great applicability. For example, there are some reports about the use of EN methods and pattern recognition (PARC) techniques for classification and quality evaluation of Medicinal and Aromatic Plants (MAPs) in the literature [10,11,12,13].

In recent years, EN systems have been widely tested for quality control of products in the food and aroma industries [14]. ENs are instruments which mimic the human olfactory perception through an array of chemical sensors (e.g., metal oxide semiconductor sensors) with partial specificity and overlapping sensitivity, combined with an appropriate PARC system for recognizing simple or complex odors [15,16]. However, this sensor technology is still far from the sensitivity and selectivity of the human nose [17]. Complex odors are evaluated by ENs as patterns or ‘‘fingerprints’’, rather than separating, identifying, and quantifying every single volatile compound present in the mixture [18,19]. In the case of the electronic olfactory systems, these patterns are the sensor array responses. From these responses, features are pre-processed and extracted for every sensor. Then, these features are used by machine learning algorithms, which allow artificial systems to infer in a non-destructive manner typical parameters in the food industry such as quality, ripeness, and shelf life [20,21,22] or to detect or identify adulterated products [23,24,25]. All of these applications are somehow related to the common goal of classifying the unknown quality of samples in a simple, fast, and effective way using an EN. In machine learning and multivariate statistics, classification consists of how to assign a new observation to a given category defined during the calibration (or training phase) of a particular application.

Classification problems can be divided into two groups: binary and multiclass problems. In binary problems, measurements need to be classified only into two classes, whereas multiclass classification involves assigning an object to one of several classes [26]. The binary problems have specific algorithms that tend to be simpler and more robust. Also, many classification methods have been developed specifically for binary problems. Using these algorithms for multiclass classification often requires the combined use of multiple binary classifiers.

**Figure 1:**
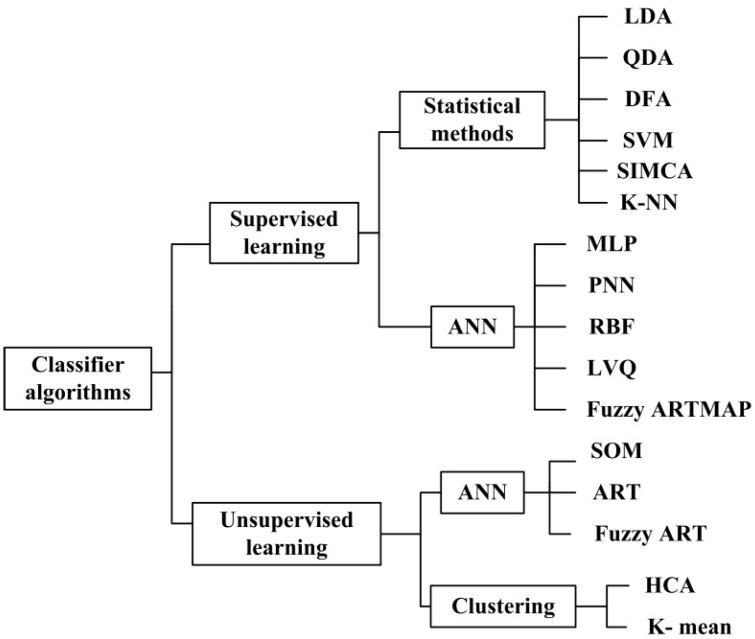
Grouping of classifier algorithms applied to electronic noses (ENs). ANN refers to Artificial Neural Network. ART refers to Adaptive Resonant Theory neural network. ARTMAP refers to Adaptive Resonant Theory Map. DFA refers to Discriminant Factor Analysis. HCA refers to Hierarchical Cluster Analysis. LDA refers to Linear Discriminant Analysis. LVQ refers to Learning Vector Quantization. K-NN refers to K Nearest Neighbors. MLP refers to Multi-Layer Perceptron. PNN refers to Probabilistic Neural Network. QDA refers to Quadratic Discriminant Analysis. RBF refers to Radial Basis Function neural network. SIMCA refers to Soft Independent Modelling of Class Analogies. SOM refers to Self-Organizing Map. SVM refers to Support Vector Machine.

Many multivariate analysis techniques have been applied to ENs for PARC analyses, which are divided typically into two categories: Supervised and unsupervised learning (see Figure 1). In supervised learning, data are divided into training and evaluation datasets during the calibration and validation phases, respectively. The training set has specified inputs (predictors or variables) and outputs (targets or classes) but the evaluation dataset has only input vectors. The PARC algorithm gives an output which is used then for model verification and evaluation giving a classification accuracy figure (validation). Unsupervised learning methods do not need *a priori* knowledge about class membership because they cluster the measurements into different classes using only the sensor matrix responses as input vectors [27].

In this paper, we envisage two goals. Initially, in order to have an independent reference method for classifying the different samples, analyzed, samples were subject to a GC-MS characterization to identify their constituents. This study was further used to define a set of different classes, based on the total percent of the most important constituents identified, according to which samples were classified. In other words, three objective quality categories were created in the first stage and each sample was assigned to one of the three categories. The second goal was to implement an EN system based on metal oxide semiconductor sensors (MOS) and to find a suitable PARC method to optimize the EN performance in the particular task of quality classification of *Rosa damascene* essential oils. Our investigation has been focused on two classifier algorithms, LDA (linear discriminant analysis) and Fuzzy ARTMAP (Adaptive Resonant Theory Map) (both PARC techniques), and on finding which subset of the features extracted from the sensor matrix responses has to be employed for improving the performance of the system.

## 2. Materials and Methods

### 2.1. Sample Preparation

In the experimentation stage, 10 genotypes of Rosa were selected and their petals were gathered from a field at the Research Institute of Forests and Rangelands (RIFR), Iran. The essential oils were extracted by hydro-distillation using a Clevenger type apparatus for 2 h [28,29]. The collected samples were stored in a dark room at 4 °C until analyzed by GC-MS.

### 2.2. GC-MS

The analysis of the volatile constituents was done on a Hewlett-Packard Agilent 6890 gas chromatograph equipped with an automatic liquid sampler (HP7683 Series) and an analytical column HP-5MS (5% phenyl methyl siloxane, 30 m length × 0.25 mm in diameter, film thickness of 0.25 μm), connected to a Hewlett-Packard mass spectrometer (5973 Agilent Technologies, Santa Clara, CA, USA). Oven temperature programming started at 80 °C for 0.5 min and then increased at the rate of 5 °C/min to 200 °C. Next, the temperature was increased to 300 °C at a rate of 15 °C/min and held for 10 min; injected volume and split ratio were 1 μL and 200:1, respectively; ionization voltage was 70 eV and the monitored mass range was 35–600 amu; helium was used as gas carrier with a flow rate of 1.2 mL·min^−1^ and the mass spectroscopy detector (MSD) transfer line temperature was 250 °C. The sample was prepared by dilution of the oil with 200 mL of dichloromethane and direct injection to the GC-MS; 200 μL of essential oil were diluted in 100 μL dichloromethane for injection.

Individual compounds were detected and identified by comparing their retention indices and recorded mass spectra with the National Institute of Standards and Technology (NIST 11.0) mass-spectral library, Wiley MS data system library (Wiley, Chichester, UK) and previous literature. The retention indices were calculated from all of the volatile constitutes using a homologous series of n-alkanes (Sigma–Aldrich Trading Co., Ltd., Shanghai, China) [30,31].

### 2.3. Electronic Nose Design and Operation

**Figure 2:**
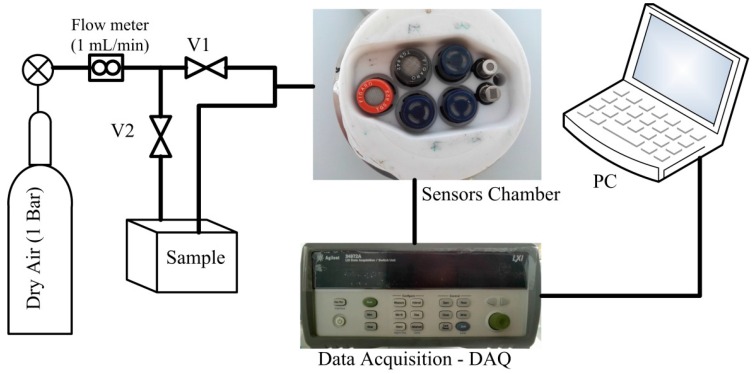
Schematics of an electronic nose based on seven metal oxide sensors (MOS).

**Figure 3:**
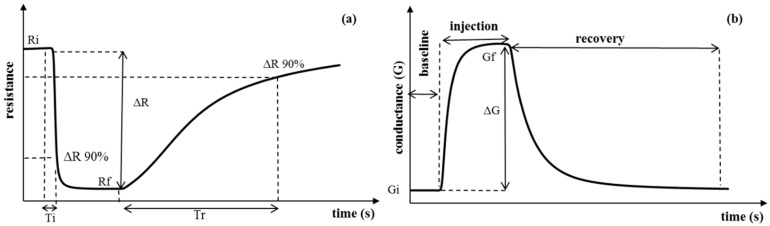
Response patterns of sensors to volatile oils, (**a**) and (**b**) represent, respectively, resistance and conductance evolution through time in the different stages of a single EN essential oil sample measurement.

An EN system was designed based on seven MOS TGS and FIS sensors [32,33]. These sensors were arranged in a chamber with 0.25 L in volume and an inlet and outlet path. The electronic circuitry schematics of the system and sensor characteristics are shown in Figure 2 and Table 1, respectively. The response pattern of sensors *(i.e.*, their resistance change) was recorded by a data acquisition card (Agilent, LXI-34972A) during three different phase periods: baseline response (60 s), injection response (200 s), and recovery response (500 s), as illustrated in Figure 3. In the baseline phase, dry air is pumped into the sensors’ chamber through valve 1 with a rate of 1 mL·min^−1^. During this phase, the sensors show a stable status. Then, their response signal changes by the injection of the headspace of the samples to the sensors’ chamber through valve 2. After the measurement, a purging phase of the sensors’ chamber employing dry air results in a recovery step in which sensors return to a stable baseline resistance. Eleven measurements were acquired every day followed by an ethanol calibration to reset the medium to its original state. Each measurement cycle lasted 760 s and the whole experimentation took 15 days to be completed.

### 2.4. PARC Techniques (LDA and Fuzzy ARTMAP)

The LDA classifier models the difference between sample categories by finding a discriminant function (DF), which is a linear combination of the original variables (features of the sensor responses) that tries to maximize the variance between classes and minimize the variance within classes [22,34]. Many applications of these techniques have been reported in the literature [35,36].

**Figure 4:**
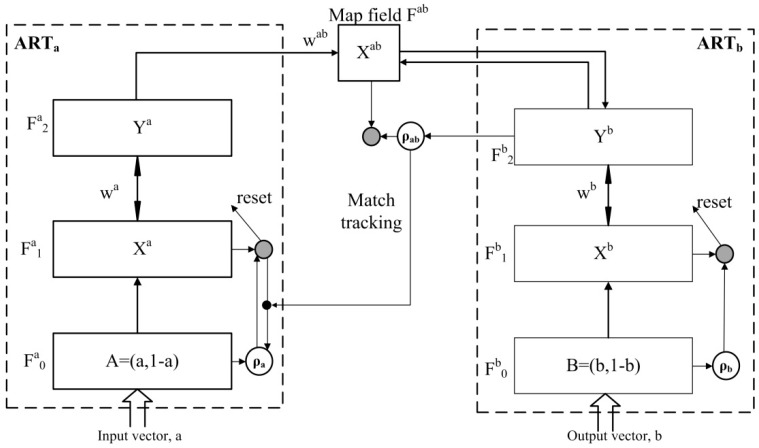
Fuzzy ARTMAP structure, a is the input vector (features obtained from the sensors’ response) and b is corresponding class to measurement described by input vector a (during training or calibration). For more information about other parameters, the reader is referred to the cited literature [27,45].

In the case of artificial neural networks (ANN) for PARC, the back-propagation multilayer perceptron (MLP) neural network has been the most widely used in EN applications [37,38]. MLP neural network has a slow learning and calculation-intensive training procedure, since a number of hidden neurons must be set by the user. Setting this value too low or too high may lead to an under fitted or over fitted model, respectively [39]. Off-line training is another important drawback since MLPs are unable to adapt autonomously, in real time, to changes in the environment [27] while measurements are done on routine operation. On the other hand, the Fuzzy ARTMAP machine learning algorithm is a self-organizing model that can be adapted or re-calibrated online with changing conditions coming from new measurements, even those made during routine operation of the instrument. Another advantage is that while in MLP networks the number of hidden layer(s) and nodes must be determined before training, the Fuzzy ARTMAP algorithm does not need the predetermination of most of its parameters such as the number of hidden neurons. Moreover, the Fuzzy ARTMAP classifier performs much faster compared to MLP networks, both during training and during evaluation, since it requires far less computations. Thus, it is appropriate for PARC functions in dynamic environments that are subjected to the presence of new patterns [40]. The fuzzy ARTMAP network has become a successful pattern recognition method for processing binary and analogue input patterns in EN applications. This is due to its special learning method characterized by the stability–plasticity dilemma, which more closely emulates human learning [41,42,43,44]. This model is based on adaptive resonance theory (ART) and Fuzzy set theory, and was introduced by Carpenter in [45]. For more details on fuzzy set theory and the ART neural network the reader is referred to the referenced literature [46,47,48]. The fuzzy ARTMAP is a supervised version of Fuzzy ART and is based on the use of two Fuzzy ART modules (ARTa and ARTb) interconnected by an associative memory (the “mapfield”, Fab) and some internal control structures as a match tracking system that regulates the complexity of the network (like the number of hidden neurons in MLPs) through the so-called vigilance parameter (ρ). Every module includes three fields, namely F0, F1, and F2, which are specified by superscripts *a* and *b* for ARTa and ARTb, respectively [45] (see Figure 4). The description of the Fuzzy ARTMAP theory is beyond the scope of this paper and therefore, for more information the reader is referred to the corresponding literature [27,45]. These characteristics confer this algorithm a great capability to learn from relatively small training sample sets that do not need to be balanced (have the same example inputs for each category outputs).

### 2.5. Statistical Analysis

One of the most important steps in classification problems is the extraction of meaningful features from raw data (e.g., the response of the sensors in EN applications). Feature extraction has been implemented employing different techniques and, clearly, choosing the appropriate features has a very important impact on the classification accuracy reached with ENs [49]. Feature extraction is often a very important aspect of the so-called data pre-processing step in EN applications. Selection of the appropriate feature extraction method heavily depends on the underlying sensor technology and the nature of any interfering signal [27]. For example, in the case of MOS, literature shows that the fractional change in conductance (Gf-Gi)/Gi helps both to linearize the sensor response *vs.* concentration and to reduce its temperature sensitivity [50,51].

As none of the extracted features alone may give a good description of the pattern generated by each sensor, seven features (five parameters based on resistance or conductance, and the other two based on response time) were extracted from every single measurement in this research. This is shown graphically in Figure 3 and a list of these features can be found in Table 2.

Since our EN comprised seven sensors, 49 features were obtained from every measurement. Fifteen replicate measurements were performed on the 10 different samples of *Rosa damascene* essential oils, and hence the database contained 150 measurements in total. All the features were scaled between 0 and 1 in order to normalize different resistance ranges and response times between several sensors. To do so, each column of the resulting matrix (each feature is a column) was divided by the maximum value present in that given column. Moreover, the Fuzzy ARTMAP algorithm needs inputs scaled between 0 and 1. In order to reduce the number of variables and select an optimal subset of features, an intra/inter variance criterion [52], also known as a Fisher ratio, according to Equation (1), was used:

(1) V_r_ = (External Variance)/(Internal Variance)

The external variance is defined as the between-category centroids’ variance and the internal variance is the average variance inside each category, which is calculated for the repetitions performed on each class. The number of classes is determined by GC-MS results so that every class may include several genotypes. A higher external variance is better since it means a good separation capability, and a lower internal variance is better because it means that the measurements in each class are closer together and there is a better reproducibility. Therefore, a higher value for Vr means better discrimination capability for a given variable [41]. After ordering features based on this criterion (from highest to lowest), the classification accuracy was calculated by LDA and Fuzzy ARTMAP classifiers with input vectors including 10, 20, 30, or 49 features. A leave-one-out cross validation (LOOCV) approach was used for testing the algorithms. This approach implies a calculation for each measurement (*i.e.*, 150 times). Each time, training is performed with 149 measurements and the remaining one is used only for evaluation purposes, so that all of measurements participate eventually in training and testing processes. This strategy maximizes the confidence about the figures of merit (the combination of PARC method and features) that each approach obtains, given the limited number of samples.

The Fuzzy ARTMAP parameters were chosen according to a strategy previously employed in the analysis of EN data [41]. The baseline vigilance parameter (ρa) was set to 0. This is the recommended value for the vigilance, since it allows for very coarse categories and the match tracking system will only refine these categories if necessary [53]. In this way, the network learns 100% of the training samples compromising to a minimum the generalization ability of the system. On the other hand, the normal way to proceed with the vigilance parameter for the ARTMAP B is to set it to 1, since the categories have to be exactly defined during training and different “label values” should be clearly separated in different ART B output neurons. The interpretation given to the network in the implementation used in this report requires the vigilance parameter for the map field ρab to be set to nearly 1 (0.99). The value of the choice parameter (α) was set to 0.001; a value well below 1 is recommended to prevent a tie outcome in competitions between neurons. The Fuzzy ARTMAP was used in fast learning mode (β = 1), meaning that a single training measurement allows the mode to create and learn a new category (hence the name “fast learning”). This is also possible because the measurements are not noisy, otherwise a slow recode procedure would have been proposed. Matlab v8.5 (The Mathworks, Natick, MA, USA) and R v3.2.1 were used for data analysis.

## 3. Results and Discussion

### 3.1. GC-MS Results

As it can be seen in Table 3, the result of the GC-MS measurements showed that numerous volatile components co-exist in an essential oil sample. In other words, the headspace (or aroma) of these types of samples is considered a complex odor with many compounds, generally in the order of hundreds or thousands. Among these components, the most influencing constituents on the quality determination of Iranian Rosa essential oil are phenyl ethyl alcohol, trans rose oxide, citronellol, nerol, geraniol, and geranial [3,29]. Therefore, the qualitative classification of samples was implemented based on the total percent concentration of the first six constituents that are specified in Table 3. A coarse and straightforward quality classification was established. Low quality (class 1) corresponded to genotypes in which the total percent of the six relevant constituents was below 10% (*i.e.*, samples labelled g1, g2, g3, and g4). Middle quality (class 2) corresponded to genotypes in which this total percent was higher than 10% and lower than 50% (*i.e.*, samples g5, g6, and g7). Finally, high quality (class 3) was attributed to samples in which the amount of the relevant constituents accounted for more than 50% of the total (g8, g9, and g10).

### 3.2. Classification Results

**Figure 5:**
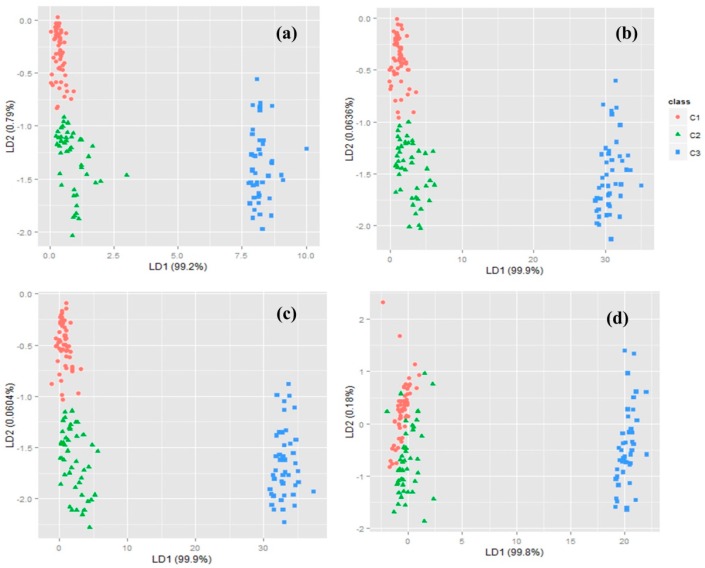
Score plots of LDA analyses based on 10 (**a**); 20 (**b**); 30 (**c**); and 49 (**d**) features.

**Figure 6:**
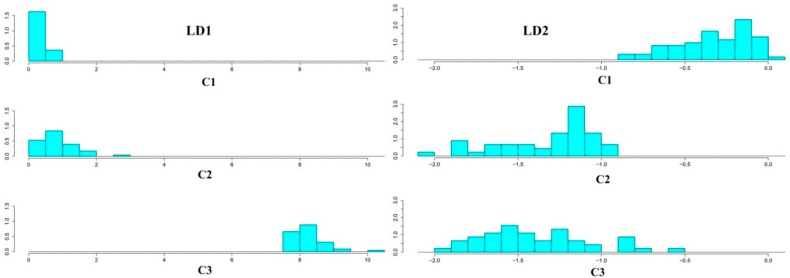
Histogram plots for first 10 features based on discriminant functions (LD1 and LD2).

The classification results of the new measurements on the coordinates based on first linear discriminant (LD1) and second linear discriminant (LD2) are shown in Figure 5a–d as score plots. These variables are linear combinations of 10, 20, 30, or 49 extracted features. According to the results, LD1 proved to separate the first and second classes (C1, C2) from class 3 (C3), but did not discriminate class 1 from class 2. On the other hand, when categorizing precisely the first and second classes, the LD2 performed better than LD1, although it could not correctly discriminate C1 and C2 when 49 features were used. Since LD2 explains the higher amount of variance when the first 10 features are used (0.8% in Figure 5a), a histogram plot was obtained for the first 10 features (Figure 6) and confirmed that each of the linear discriminant functions (LD1 and LD2) could not correctly classify all categories alone. The results of classification accuracy and confusion matrixes for the selected features are shown in Table 4. Regarding the confusion matrices, each column specifies the predicted category of each sample by the algorithm and each row specifies the actual number of samples that were tested by the EN. According to Table 4, the classification accuracy reached by the LDA method is relatively low, since the number of misclassified samples is rather high, especially for C2. Therefore, our study shows that LDA performs poorly in this 3-category classification. According to these results, the success rate of classification decreases from 82% to 62% when the number of selected features increases from 10 to 49 in LDA analysis. Therefore, the highest success rate was achieved when selecting the best 10 variables according to the Vr parameter. These first 10 selected variables ae listed in Table 5. The results show that the best features extracted from sensor responses are based on f5, f7, f3, and f4 (listed in Table 2). f5, in particular, is very effective in the classification process, since the first five variables selected are feature 5 for five different sensors. Also, sensors S1, S6, S7, S4, and S3 seem to be more informative than S2 and S5 for sample classification. Taking into consideration that effective quality components in Iranian Rosa are alcohols and terpenoids, sensors more sensitive to these components would be more advantageous in the classification process, as is the case.

The classification results using the Fuzzy ARTMAP neural paradigm are also shown in Table 4. According to the results, the success rate increases to 99% when the best 10 variables were selected based on the Vr parameter. Therefore, the Fuzzy ARTMAP algorithm is very successful at performing the 3-category classification of essential oil samples employing EN response patterns. The number of misclassified samples is very low and remains quite stable, no matter the number of features used. For example, there is only one misclassified measurement when 10 features are selected. It is well-known that the response of MOS is rather nonlinear and the fact that the Fuzzy ARTMAP learning paradigm is more suited to deal with non-linear data than LDA (which is a linear method) could explain the better results obtained by the former. Additionally, experimental data are subject to some degree of uncertainty (e.g., due to noise) and the Fuzzy ARTMAP is also more suited to deal with noise [27]. Finally, the Fuzzy ARTMAP has stable learning and is able to carry out on-line learning without forgetting previously learnt patterns, which helps this method to be quite resilient to the presence of sensor drift [27].

## 4. Conclusions

The chemical constituents of Iranian *Rosa damascene* essential oils were characterized by GC-MS analysis. According to these results, the samples were divided into three qualitative categories (low, middle, and high quality) based on the total percent of six constituents that are known to be relevant in the determination of the quality of the essential oil of *Rosa damascene*. The algorithms Fuzzy ARTMAP and linear discriminant analysis (LDA) were used to classify samples of essential oils from *Rosa damascene* by an electronic nose system whose design was based on seven metal oxide sensors. When the samples were measured by the EN, 49 features were extracted from the sensor matrix response patterns and ordered (from highest to lowest) by an intra/inter variance criterion. Then, classification accuracy was calculated when the best 10, 20, 30, or 49 features extracted were used for training and validating Fuzzy ARTMAP and LDA algorithms via a leave-one-out approach. The best success rates obtained were 99% and 82% for Fuzzy ARTMAP and LDA analysis, respectively. These rates were obtained when the best 10 features were input to the classifiers in both cases. These results show that Fuzzy ARTMAP performs much better than LDA in all feature selection scenarios. Therefore, Fuzzy ARTMAP is a much better technique for pattern recognition and classification of Rosa essential oils measured with an electronic nose. On the other hand, for both techniques, the best results were achieved when only the best 10 features were selected, meaning that a more parsimonious (simple) PARC model with fewer input variables achieves a better generalization performance than a more complicated model. Therefore, we conclude that an EN can be used as an easy, low cost, accurate, and rapid method for the qualitative classification of Iranian *Rosa damascene* essential oils.

## Figures and Tables

**Table 1:** Sensor array used in the electronic nose system.

Sensor Number	Sensor Name	Target Gas
S1	TGS *-822	Organic Solvent Vapors
S2	TGS-842	Methane
S3	SP **-15A	LP gas (butane-propane)
S4	SP-32	Alcohol
S5	SP-53	Ammonia
S6	TGS-2610	LP gas (butane-Propane)
S7	TGS-2620	Organic Solvent Vapors

* Figaro Engineering (Osaka, Japan); ** FIS (Hyogo, Japan).

**Table 2:** Features extracted from sensors response.

Features	Calculation
f1	$\frac{R_{i}}{R_{f}}$
f2	$\Delta R=R_{i}-R_{f}$
f3	$\frac{R_{i}-R_{f}}{R_{i}}$
f4	$\Delta G=G_{f}-G_{i}$
f5	$\frac{G_{f}-G_{i}}{G_{i}}$
f6	$T_{i}=t_{0.9\Delta R}-t_{R_{i}}$
f7	$T_{r}=t_{0.9\Delta R}-t_{R_{f}}$

**Table 3:** Chemical constituents of Iranian Rosa essential oils determined by GC-MS analysis.

No.	Cons. Name	Formula	Constituent Percentage for Each Genotype									
			g10	g9	g8	g7	g6	g5	g4	g3	g2	g1
1	**Phenyl ethyl alcohol**	**C_8_H_10_O**	**0.64**	**0.18**	**0.61**	**0.37**	**0.3**	**0.3**	**0.21**	**-**	**-**	**-**
2	**Trans rose oxide**	**C_10_H_18_O**	**1.25**	**0.61**	**0.5**	**0.61**	**0.4**	**0.23**	**0.12**	**0.32**	**0.18**	**0.4**
3	**Citronellol**	**C_10_H_20_O**	**28.18**	**53.1**	**33.7**	**24.1**	**13.35**	**8.48**	**2.14**	**4.73**	**0.48**	**1.25**
4	**Nerol**	**C_10_H_18_O**	**16.2**	**2.01**	**3.56**	**0.1**	**0.1**	**0.6**	**-**	**-**	**-**	**-**
5	**Geraniol**	**C_10_H_18_O**	**30.02**	**15.16**	**26.05**	**12.1**	**7.25**	**6.15**	**6.33**	**2.66**	**3.25**	**0.5**
6	**Geranial**	**C_10_H_16_O**	**0.48**	**0.26**	**0.27**	**0.1**	**0.21**	**0.2**	**0.1**	**0.14**	**0.15**	**-**
7	α-Eudesmol	C_15_H_26_O	0.72	-	0.61	-	3.15	2.18	-	2.35	2.86	1.73
8	β-Eudesmol	C_15_H_26_O	-	-	0.67	-	2.84	2.52	2.01	2.77	4.33	1.9
9	γ-Eudesmol	C_15_H_26_O	-	-	0.57	-	2.68	2.43	-	1.4	1.77	1.48
10	Cyclohexanemethanol	C_15_H_26_O	-	-	-	-	1.25	0.53	-	0.17	0.62	0.44
11	Dioctyl Phthalate	C_24_H_38_O_4_	-	-	-	-	-	-	-	-	-	15.9
12	Farnesol	C_15_H_26_O	-	-	0.27	1.53	-	-	-	0.9	0.95	-
13	Octyl phthalate	C_24_H_38_O_4_	2.48	-	0.28	-	-	0.22	3.91	-	12.0	-
14	Geranyl acetate	C_12_H_20_O_2_	2.05	0.44	1.12	1.24	0.71	-	0.5	0.1	-	-
15	Methyleugenol	C_11_H_14_O_2_	-	0.28	0.25	0.75	-	-	-	0.15	-	-
16	Diisooctyl phthalate	C_24_H_38_O_4_	-	5.16	-	-	-	-	-	4.88	-	-
17	Linalool	C_10_H_18_O	-	-	0.53	0.31	-	-	0.83	-	-	-
18	Neral	C_10_H_16_O	1.3	1.0	0.94	0.9	0.78	0.7	0.66	0.34	0.23	0.1
19	3-Methyl-4-isopropylphenol	C_10_H_14_O	-	0.53	-	0.3	-	-	-	-	-	-
20	Eugenol	C_10_H_12_O_2_	-	-	-	0.4	-	-	-	-	-	-
21	Apilo	C_12_H_14_O_4_	-	-	0.23	0.42	-	-	-	-	-	-
22	Nonacosane	C_29_H_6_O	-	-	0.37	0.56	-	-	-	-	-	-
23	Nonanal	C_9_H_18_O	-	-	0.13	-	-	-	-	-	-	-
24	Anethole	C_10_H_12_O	-	0.55	0.5	-	-	-	-	-	-	-
25	Chavibetol	C_10_H_12_O_2_	-	0.22	0.28	-	-	-	-	-	-	-
26	Docosane	C_22_H_46_	-	3.82	4.0	0.53	4.05	4.83	6.51	5.0	0.55	-
27	Pentacosane	C_25_H_52_	-	2.25	-	2.06	1.65	-	-	-	2.44	-
28	z-5-Nonadecene	C_19_H_38_	-	2.93	-	-	-	6.35	6.33	6.35	5.35	5.6
29	Nonadecane	C_19_H_40_	10.94	12.76	14.2	17.6	35.9	40.3	37.2	33.7	30.5	33.1
30	Eicosane	C_20_H_42_	1.9	2.48	2.28	3.12	2.73	3.76	4.15	5.4	3.43	3.28
31	Hexadecane	C_20_H_42_	-	-	-	-	-	-	-	-	-	4.48
32	1-Tetradecene	C_14_H_28_	-	-	-	-	-	-	-	0.13	-	-
33	9-Eicosene	C_20_H_40_	-	-	0.2	-	-	-	-	0.18	-	-
34	9-Nonadecene	C_19_H_38_	-	0.21	0.33	-	-	-	0.35	0.37	-	-
35	cis-9-Tricosene	C_23_H_46_	-	0.46	-	0.55	-	-	-	0.28	-	-
36	Bicyclo[10.8.0]eicosane-cis	C_20_H_38_	-	-	-	-	-	-	-	-	-	0.13
37	Hexacosane	C_26_H_54_	-	-	-	-	-	0.2	-	0.2	-	-
38	Octacosane	C_28_H_58_	-	-	0.09	-	-	-	-	0.07	-	-
39	Heneicosane	C_21_H_44_	10.0	9.66	11.2	11.6	17.34	20.7	25.7	19.6	20.2	20.0
40	Tetracosane	C_24_H_50_	0.5	2.6	0.33	0.41	-	2.42	0.27	0.6	3.68	2.87
41	Neopentylidenecyclohexane	C_11_H_20_	-	-	-	-	-	1.35	-	-	-	-
42	1,21-Docosadiene	C_22_H_42_	-	-	-	-	-	0.15	-	-	-	-
43	1-Octadecene	C_18_H_36_	-	-	-	-	6.03	-	-	-	-	-
44	8-Heptadecan	C_17_H_34_	-	-	0.35	-	1.5	1.84	-	1.16	0.86	0.52
45	2,6-Octadiene, 2,6-dimethyl	C_10_H_18_	-	0.36	0.37	0.35	-	-	-	-	-	-
46	Heptacosane	C_27_H_56_	-	0.24	2.4	2.13	2.0	0.07	3.37	2.66	-	2.91
47	Bergamoten	C_15_H_24_	-	-	-	0.84	-	-	-	-	-	-
48	Teriacontane	C_30_H_62_	1.78	0.4	-	-	-	-	-	-	-	0.31
49	1-Nonadecane	C_19_H_38_	-	-	-	3.5	-	-	-	-	-	-
50	Tricosane	C_23_H_48_	-	-	-	3.75	-	-	-	-	-	-
51	1,19-Eicosadiene	C_20_H_38_	-	-	0.16	-	-	-	-	-	-	-
52	5-Eicosene, (E)	C_20_H_40_	3.96	-	-	-	-	-	-	-	-	-
53	Pentadecane	C_15_H_32_	-	0.21	0.21	-	0.28	-	-	0.4	-	0.36
54	Heptadecane	C_17_H_36_	-	1.6	1.78	1.56	2.1	3.67	2.51	3	2.17	2.93
55	7-Tetradecyne	C_14_H_26_	-	-	-	-	-	-	-	-	-	0.4
56	Octadecane	C_18_H_38_	-	0.6	2.16	0.4	-	0.4	2.68	0.27	5.0	0.25
**Total Percent for first six constituents**			**76.77**	**71.32**	**64.69**	**37.4**	**21.61**	**15.96**	**8.9**	**7.85**	**4.06**	**2.15**

**Table 4:** Success rates in classification and confusion matrices for selected features by LDA and Fuzzy ARTMAP.

	Analysis									
	LDA					Fuzzy ARTMAP				
No. Features	Real	Predicted			Success Rate	Real	Predicted			Success Rate
		C1	C2	C3			C1	C2	C3	
	**C1**	60	0	0		**C1**	59	1	0	
**10**	**C2**	18	17	10	82%	**C2**	0	45	0	99%
	**C3**	0	0	45		**C3**	0	0	45	
	**C1**	59	0	1		**C1**	58	2	0	
**20**	**C2**	21	11	13	73%	**C2**	0	44	1	98%
	**C3**	6	0	39		**C3**	0	0	45	
	**C1**	57	0	3		**C1**	58	2	0	
**30**	**C2**	23	10	12	67%	**C2**	0	43	2	97%
	**C3**	12	0	33		**C3**	0	0	45	
	**C1**	57	0	3		**C1**	58	2	0	
**49**	**C2**	25	7	13	62%	**C2**	1	41	3	96%
	**C3**	16	0	29		**C3**	0	0	45	

**Table 5:** The first 10 variables selected.

Variables NO.	Variables Name
1	S1f5
2	S6f5
3	S7f5
4	S4f5
5	S3f5
6	S3f7
7	S4f7
8	S3f3
9	S4f3
10	S6f4

## Abbreviations

The following abbreviations are used in this manuscript:

ANN	Artificial Neural Network
ART	Adaptive Resonant Theory neural network
ARTMAP	Adaptive Resonant Theory Map
DFA	Discriminant Factor Analysis
EN	Electronic Nose
GC-MS	Gas Chromatography coupled to Mass Spectrometry
HCA	Hierarchical Cluster Analysis
HIV	Human Immunodeficiency Virus
HPLC	High Precision Liquid Chromatography
K-NN	K Nearest Neighbors
LDA	Linear Discriminant Analysis
LOOCV	Leave One Out Cross Validation
LVQ	Learning Vector Quantization
MAP	Medicinal and Aromatic Plant
MLP	Multi-Layer Perceptron
MOS	Metal Oxide Sensor
MSD	Mass Spectroscopy Detector
PARC	Pattern Recognition
PNN	Probabilistic Neural Network
QDA	Quadratic Discriminant Analysis
RBF	Radial Basis Function neural network
SIMCA	Soft Independent Modelling of Class Analogies
SOM	Self-Organizing Map
SVM	Support Vector Machine
TLC	Thin Layer Chromatography
